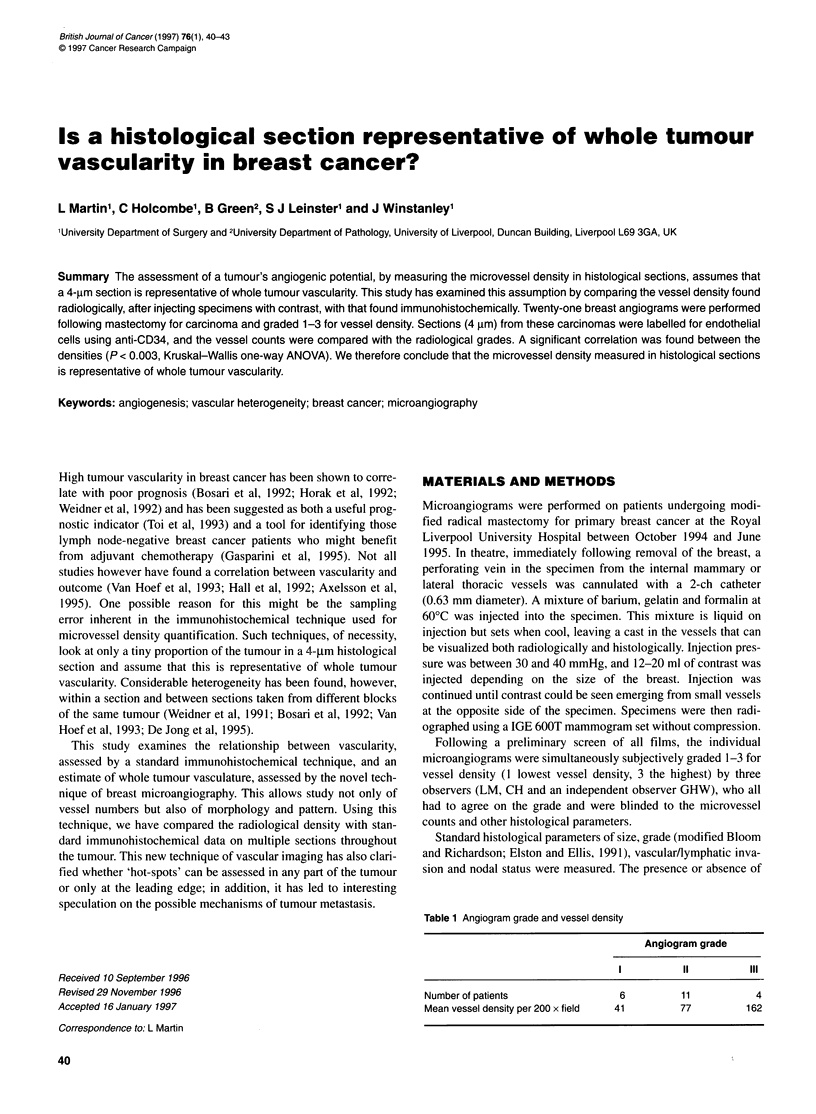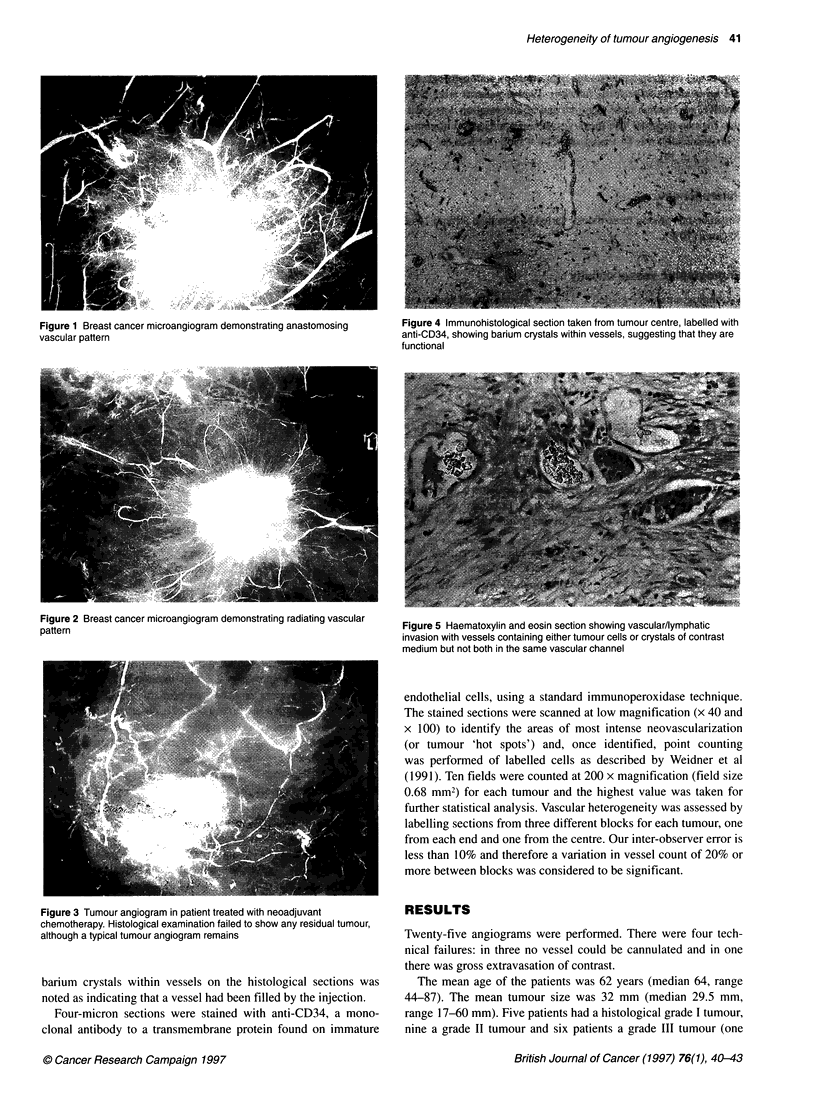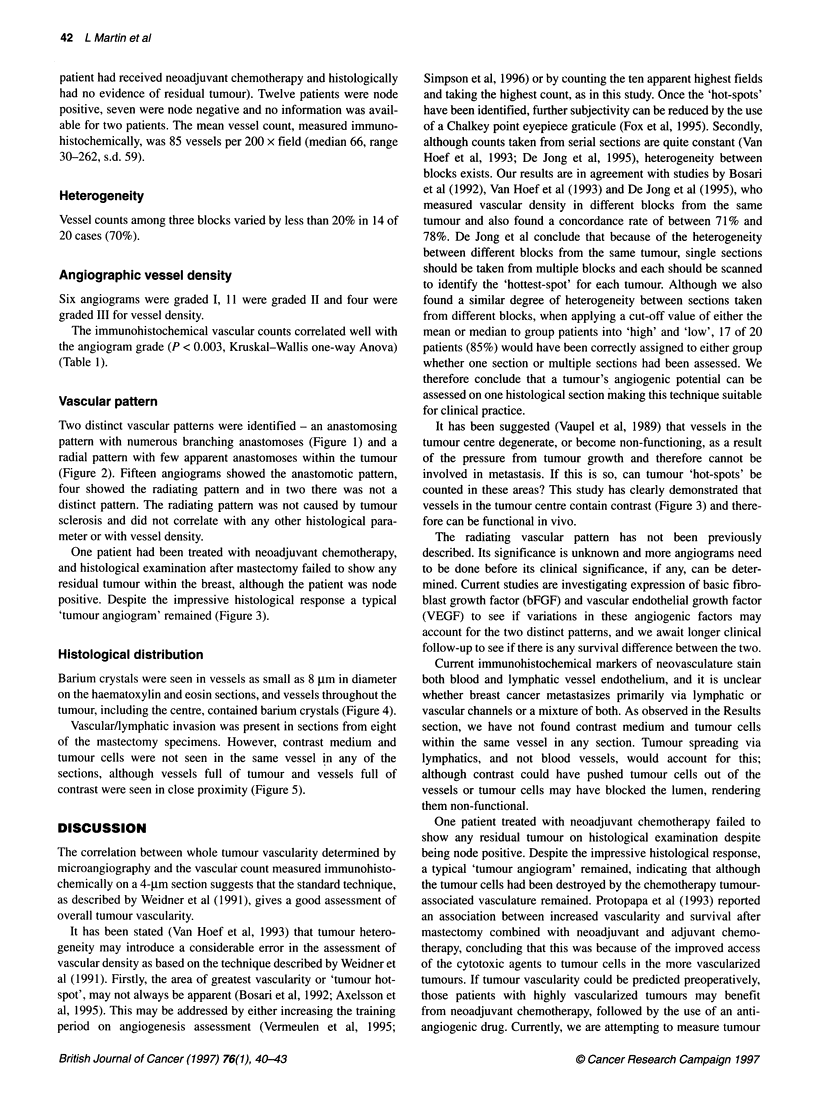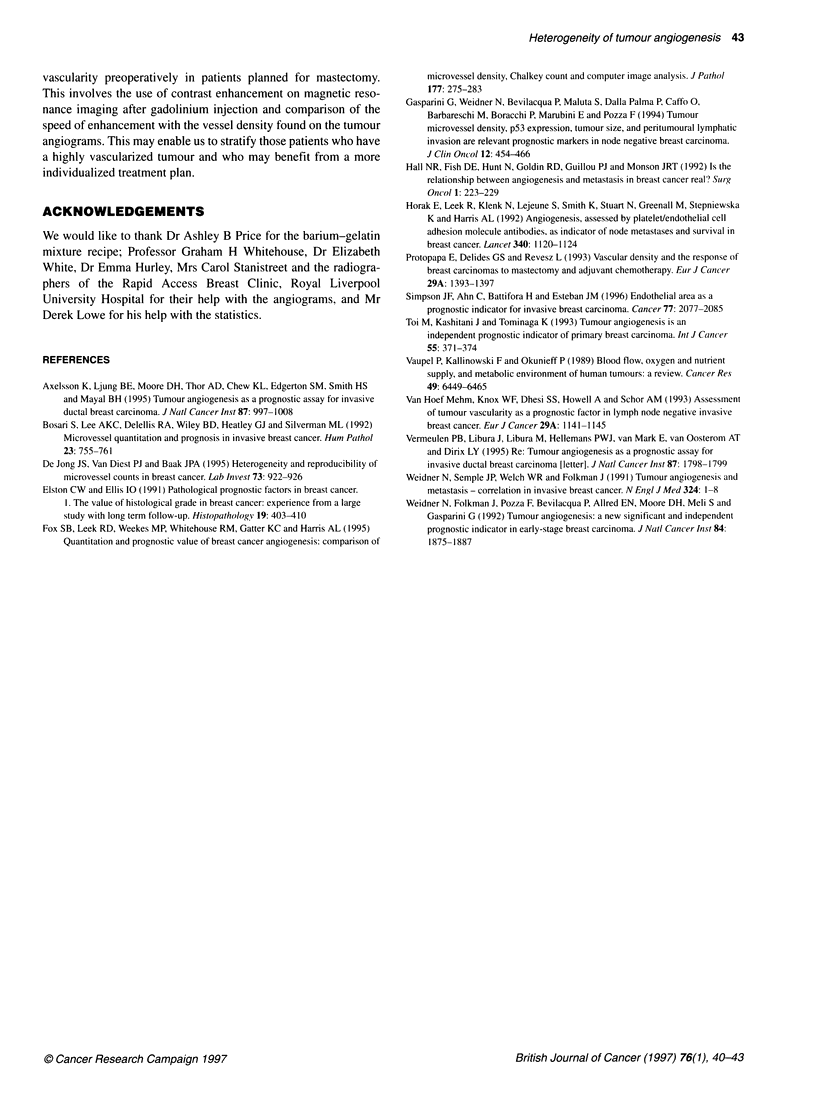# Is a histological section representative of whole tumour vascularity in breast cancer?

**DOI:** 10.1038/bjc.1997.333

**Published:** 1997

**Authors:** L. Martin, C. Holcombe, B. Green, S. J. Leinster, J. Winstanley

**Affiliations:** University Department of Surgery, University of Liverpool, UK.

## Abstract

**Images:**


British Journal of Cancer (1997) 76(1), 40–43
© 1997 Cancer Research Campaign

### Is a histological section representative of whole tumour vascularity in breast cancer?

**L Martin[1], C Holcombe[1], B Green[2], S J Leinster[1] and J Winstanley[1]**

[1]University Department of Surgery and [2]University Department of Pathology, University of Liverpool, Duncan Building, Liverpool L69 3GA, UK

**Summary** The assessment of a tumour's angiogenic potential, by measuring the microvessel density in histological sections, assumes that a 4-μm section is representative of whole tumour vascularity. This study has examined this assumption by comparing the vessel density found radiologically, after injecting specimens with contrast, with that found immunohistochemically. Twenty-one breast angiograms were performed following mastectomy for carcinoma and graded 1–3 for vessel density. Sections (4 μm) from these carcinomas were labelled for endothelial cells using anti-CD34, and the vessel counts were compared with the radiological grades. A significant correlation was found between the densities ($P < 0.003$, Kruskal–Wallis one-way ANOVA). We therefore conclude that the microvessel density measured in histological sections is representative of whole tumour vascularity.

**Keywords:** angiogenesis; vascular heterogeneity; breast cancer; microangiography

High tumour vascularity in breast cancer has been shown to correlate with poor prognosis (Bosari et al, 1992; Horak et al, 1992; Weidner et al, 1992) and has been suggested as both a useful prognostic indicator (Toi et al, 1993) and a tool for identifying those lymph node-negative breast cancer patients who might benefit from adjuvant chemotherapy (Gasparini et al, 1995). Not all studies however have found a correlation between vascularity and outcome (Van Hoef et al, 1993; Hall et al, 1992; Axelsson et al, 1995). One possible reason for this might be the sampling error inherent in the immunohistochemical technique used for microvessel density quantification. Such techniques, of necessity, look at only a tiny proportion of the tumour in a 4-μm histological section and assume that this is representative of whole tumour vascularity. Considerable heterogeneity has been found, however, within a section and between sections taken from different blocks of the same tumour (Weidner et al, 1991; Bosari et al, 1992; Van Hoef et al, 1993; De Jong et al, 1995).

This study examines the relationship between vascularity, assessed by a standard immunohistochemical technique, and an estimate of whole tumour vasculature, assessed by the novel technique of breast microangiography. This allows study not only of vessel numbers but also of morphology and pattern. Using this technique, we have compared the radiological density with standard immunohistochemical data on multiple sections throughout the tumour. This new technique of vascular imaging has also clarified whether 'hot-spots' can be assessed in any part of the tumour or only at the leading edge; in addition, it has led to interesting speculation on the possible mechanisms of tumour metastasis.

*Received 10 September 1996*
*Revised 29 November 1996*
*Accepted 16 January 1997*

*Correspondence to:* L Martin

### MATERIALS AND METHODS

Microangiograms were performed on patients undergoing modified radical mastectomy for primary breast cancer at the Royal Liverpool University Hospital between October 1994 and June 1995. In theatre, immediately following removal of the breast, a perforating vein in the specimen from the internal mammary or lateral thoracic vessels was cannulated with a 2-ch catheter (0.63 mm diameter). A mixture of barium, gelatin and formalin at 60°C was injected into the specimen. This mixture is liquid on injection but sets when cool, leaving a cast in the vessels that can be visualized both radiologically and histologically. Injection pressure was between 30 and 40 mmHg, and 12–20 ml of contrast was injected depending on the size of the breast. Injection was continued until contrast could be seen emerging from small vessels at the opposite side of the specimen. Specimens were then radiographed using a IGE 600T mammogram set without compression.

Following a preliminary screen of all films, the individual microangiograms were simultaneously subjectively graded 1–3 for vessel density (1 lowest vessel density, 3 the highest) by three observers (LM, CH and an independent observer GHW), who all had to agree on the grade and were blinded to the microvessel counts and other histological parameters.

Standard histological parameters of size, grade (modified Bloom and Richardson; Elston and Ellis, 1991), vascular/lymphatic invasion and nodal status were measured. The presence or absence of

**Table 1** Angiogram grade and vessel density

| | Angiogram grade | | |
|---|---|---|---|
| | I | II | III |
| Number of patients | 6 | 11 | 4 |
| Mean vessel density per 200 × field | 41 | 77 | 162 |

**Figure 1** Breast cancer microangiogram demonstrating anastomosing vascular pattern

**Figure 2** Breast cancer microangiogram demonstrating radiating vascular pattern

**Figure 3** Tumour angiogram in patient treated with neoadjuvant chemotherapy. Histological examination failed to show any residual tumour, although a typical tumour angiogram remains barium crystals within vessels on the histological sections was noted as indicating that a vessel had been filled by the injection.

Four-micron sections were stained with anti-CD34, a monoclonal antibody to a transmembrane protein found on immature

**Figure 4** Immunohistological section taken from tumour centre, labelled with anti-CD34, showing barium crystals within vessels, suggesting that they are functional

**Figure 5** Haematoxylin and eosin section showing vascular/lymphatic invasion with vessels containing either tumour cells or crystals of contrast medium but not both in the same vascular channel endothelial cells, using a standard immunoperoxidase technique. The stained sections were scanned at low magnification (× 40 and × 100) to identify the areas of most intense neovascularization (or tumour 'hot spots') and, once identified, point counting was performed of labelled cells as described by Weidner et al (1991). Ten fields were counted at 200 × magnification (field size 0.68 $mm^2$) for each tumour and the highest value was taken for further statistical analysis. Vascular heterogeneity was assessed by labelling sections from three different blocks for each tumour, one from each end and one from the centre. Our inter-observer error is less than 10% and therefore a variation in vessel count of 20% or more between blocks was considered to be significant.

### RESULTS

Twenty-five angiograms were performed. There were four technical failures: in three no vessel could be cannulated and in one there was gross extravasation of contrast.

The mean age of the patients was 62 years (median 64, range 44–87). The mean tumour size was 32 mm (median 29.5 mm, range 17–60 mm). Five patients had a histological grade I tumour, nine a grade II tumour and six patients a grade III tumour (one

© Cancer Research Campaign 1997

patient had received neoadjuvant chemotherapy and histologically had no evidence of residual tumour). Twelve patients were node positive, seven were node negative and no information was available for two patients. The mean vessel count, measured immunohistochemically, was 85 vessels per 200 × field (median 66, range 30–262, s.d. 59).

### Heterogeneity

Vessel counts among three blocks varied by less than 20% in 14 of 20 cases (70%).

### Angiographic vessel density

Six angiograms were graded I, 11 were graded II and four were graded III for vessel density.

The immunohistochemical vascular counts correlated well with the angiogram grade ($P < 0.003$, Kruskal–Wallis one-way Anova) (Table 1).

### Vascular pattern

Two distinct vascular patterns were identified – an anastomosing pattern with numerous branching anastomoses (Figure 1) and a radial pattern with few apparent anastomoses within the tumour (Figure 2). Fifteen angiograms showed the anastomotic pattern, four showed the radiating pattern and in two there was not a distinct pattern. The radiating pattern was not caused by tumour sclerosis and did not correlate with any other histological parameter or with vessel density.

One patient had been treated with neoadjuvant chemotherapy, and histological examination after mastectomy failed to show any residual tumour within the breast, although the patient was node positive. Despite the impressive histological response a typical 'tumour angiogram' remained (Figure 3).

### Histological distribution

Barium crystals were seen in vessels as small as 8 μm in diameter on the haematoxylin and eosin sections, and vessels throughout the tumour, including the centre, contained barium crystals (Figure 4).

Vascular/lymphatic invasion was present in sections from eight of the mastectomy specimens. However, contrast medium and tumour cells were not seen in the same vessel in any of the sections, although vessels full of tumour and vessels full of contrast were seen in close proximity (Figure 5).

### DISCUSSION

The correlation between whole tumour vascularity determined by microangiography and the vascular count measured immunohistochemically on a 4-μm section suggests that the standard technique, as described by Weidner et al (1991), gives a good assessment of overall tumour vascularity.

It has been stated (Van Hoef et al, 1993) that tumour heterogeneity may introduce a considerable error in the assessment of vascular density as based on the technique described by Weidner et al (1991). Firstly, the area of greatest vascularity or 'tumour hot-spot', may not always be apparent (Bosari et al, 1992; Axelsson et al, 1995). This may be addressed by either increasing the training period on angiogenesis assessment (Vermeulen et al, 1995; Simpson et al, 1996) or by counting the ten apparent highest fields and taking the highest count, as in this study. Once the 'hot-spots' have been identified, further subjectivity can be reduced by the use of a Chalkey point eyepiece graticule (Fox et al, 1995). Secondly, although counts taken from serial sections are quite constant (Van Hoef et al, 1993; De Jong et al, 1995), heterogeneity between blocks exists. Our results are in agreement with studies by Bosari et al (1992), Van Hoef et al (1993) and De Jong et al (1995), who measured vascular density in different blocks from the same tumour and also found a concordance rate of between 71% and 78%. De Jong et al conclude that because of the heterogeneity between different blocks from the same tumour, single sections should be taken from multiple blocks and each should be scanned to identify the 'hottest-spot' for each tumour. Although we also found a similar degree of heterogeneity between sections taken from different blocks, when applying a cut-off value of either the mean or median to group patients into 'high' and 'low', 17 of 20 patients (85%) would have been correctly assigned to either group whether one section or multiple sections had been assessed. We therefore conclude that a tumour's angiogenic potential can be assessed on one histological section making this technique suitable for clinical practice.

It has been suggested (Vaupel et al, 1989) that vessels in the tumour centre degenerate, or become non-functioning, as a result of the pressure from tumour growth and therefore cannot be involved in metastasis. If this is so, can tumour 'hot-spots' be counted in these areas? This study has clearly demonstrated that vessels in the tumour centre contain contrast (Figure 3) and therefore can be functional in vivo.

The radiating vascular pattern has not been previously described. Its significance is unknown and more angiograms need to be done before its clinical significance, if any, can be determined. Current studies are investigating expression of basic fibroblast growth factor (bFGF) and vascular endothelial growth factor (VEGF) to see if variations in these angiogenic factors may account for the two distinct patterns, and we await longer clinical follow-up to see if there is any survival difference between the two.

Current immunohistochemical markers of neovasculature stain both blood and lymphatic vessel endothelium, and it is unclear whether breast cancer metastasizes primarily via lymphatic or vascular channels or a mixture of both. As observed in the Results section, we have not found contrast medium and tumour cells within the same vessel in any section. Tumour spreading via lymphatics, and not blood vessels, would account for this; although contrast could have pushed tumour cells out of the vessels or tumour cells may have blocked the lumen, rendering them non-functional.

One patient treated with neoadjuvant chemotherapy failed to show any residual tumour on histological examination despite being node positive. Despite the impressive histological response, a typical 'tumour angiogram' remained, indicating that although the tumour cells had been destroyed by the chemotherapy tumour-associated vasculature remained. Protopapa et al (1993) reported an association between increased vascularity and survival after mastectomy combined with neoadjuvant and adjuvant chemotherapy, concluding that this was because of the improved access of the cytotoxic agents to tumour cells in the more vascularized tumours. If tumour vascularity could be predicted preoperatively, those patients with highly vascularized tumours may benefit from neoadjuvant chemotherapy, followed by the use of an anti-angiogenic drug. Currently, we are attempting to measure tumour

© Cancer Research Campaign 1997

vascularity preoperatively in patients planned for mastectomy. This involves the use of contrast enhancement on magnetic resonance imaging after gadolinium injection and comparison of the speed of enhancement with the vessel density found on the tumour angiograms. This may enable us to stratify those patients who have a highly vascularized tumour and who may benefit from a more individualized treatment plan.

### ACKNOWLEDGEMENTS

We would like to thank Dr Ashley B Price for the barium–gelatin mixture recipe; Professor Graham H Whitehouse, Dr Elizabeth White, Dr Emma Hurley, Mrs Carol Stanistreet and the radiographers of the Rapid Access Breast Clinic, Royal Liverpool University Hospital for their help with the angiograms, and Mr Derek Lowe for his help with the statistics.

### REFERENCES

Axelsson K, Ljung BE, Moore DH, Thor AD, Chew KL, Edgerton SM, Smith HS and Mayal BH (1995) Tumour angiogenesis as a prognostic assay for invasive ductal breast carcinoma. *J Natl Cancer Inst* **87**: 997–1008

Bosari S, Lee AKC, Delellis RA, Wiley BD, Heatley GJ and Silverman ML (1992) Microvessel quantitation and prognosis in invasive breast cancer. *Hum Pathol* **23**: 755–761

De Jong JS, Van Diest PJ and Baak JPA (1995) Heterogeneity and reproducibility of microvessel counts in breast cancer. *Lab Invest* **73**: 922–926

Elston CW and Ellis IO (1991) Pathological prognostic factors in breast cancer. 1. The value of histological grade in breast cancer: experience from a large study with long term follow-up. *Histopathology* **19**: 403–410

Fox SB, Leek RD, Weekes MP, Whitehouse RM, Gatter KC and Harris AL (1995) Quantitation and prognostic value of breast cancer angiogenesis: comparison of microvessel density, Chalkey count and computer image analysis. *J Pathol* **177**: 275–283

Gasparini G, Weidner N, Bevilacqua P, Maluta S, Dalla Palma P, Caffo O, Barbareschi M, Boracchi P, Marubini E and Pozza F (1994) Tumour microvessel density, p53 expression, tumour size, and peritumoural lymphatic invasion are relevant prognostic markers in node negative breast carcinoma. *J Clin Oncol* **12**: 454–466

Hall NR, Fish DE, Hunt N, Goldin RD, Guillou PJ and Monson JRT (1992) Is the relationship between angiogenesis and metastasis in breast cancer real? *Surg Oncol* **1**: 223–229

Horak E, Leek R, Klenk N, Lejeune S, Smith K, Stuart N, Greenall M, Stepniewska K and Harris AL (1992) Angiogenesis, assessed by platelet/endothelial cell adhesion molecule antibodies, as indicator of node metastases and survival in breast cancer. *Lancet* **340**: 1120–1124

Protopapa E, Delides GS and Revesz L (1993) Vascular density and the response of breast carcinomas to mastectomy and adjuvant chemotherapy. *Eur J Cancer* **29A**: 1393–1397

Simpson JF, Ahn C, Battifora H and Esteban JM (1996) Endothelial area as a prognostic indicator for invasive breast carcinoma. *Cancer* **77**: 2077–2085

Toi M, Kashitani J and Tominaga K (1993) Tumour angiogenesis is an independent prognostic indicator of primary breast carcinoma. *Int J Cancer* **55**: 371–374

Vaupel P, Kallinowski F and Okunieff P (1989) Blood flow, oxygen and nutrient supply, and metabolic environment of human tumours: a review. *Cancer Res* **49**: 6449–6465

Van Hoef Mehm, Knox WF, Dhesi SS, Howell A and Schor AM (1993) Assessment of tumour vascularity as a prognostic factor in lymph node negative invasive breast cancer. *Eur J Cancer* **29A**: 1141–1145

Vermeulen PB, Libura J, Libura M, Hellemans PWJ, van Mark E, van Oosterom AT and Dirix LY (1995) Re: Tumour angiogenesis as a prognostic assay for invasive ductal breast carcinoma [letter]. *J Natl Cancer Inst* **87**: 1798–1799

Weidner N, Semple JP, Welch WR and Folkman J (1991) Tumour angiogenesis and metastasis – correlation in invasive breast cancer. *N Engl J Med* **324**: 1–8

Weidner N, Folkman J, Pozza F, Bevilacqua P, Allred EN, Moore DH, Meli S and Gasparini G (1992) Tumour angiogenesis: a new significant and independent prognostic indicator in early-stage breast carcinoma. *J Natl Cancer Inst* **84**: 1875–1887

© Cancer Research Campaign 1997